# Experimental Study of Mechanical Properties of Polypropylene Random Copolymer and Rice-Husk-Based Biocomposite by Using Nanoindentation

**DOI:** 10.3390/ma15051956

**Published:** 2022-03-06

**Authors:** Fahad Ali Rabbani, Saima Yasin, Tanveer Iqbal, Ujala Farooq

**Affiliations:** 1 Department of Chemical, Polymer & Composite Materials Engineering, University of Engineering and Technology, Lahore (New Campus), Kala Shah Kaku 39020, Pakistan; rabbanifahad@uet.edu.pk (F.A.R.); tanveer@uet.edu.pk (T.I.); 2 Department of Chemical Engineering, University of Engineering and Technology, Lahore 39161, Pakistan; drsaima@uet.edu.pk; 3 Department of Aerospace Structures and Materials, Faculty of Aerospace Engineering, Delft University of Technology, Kluyverweg 1, 2629 HS Delft, The Netherlands

**Keywords:** nanoindentation, biocomposite, polypropylene random copolymer, rice husk, hardness, modulus, fiber reinforced composite

## Abstract

Nanoindentation is widely used to investigate the surface-mechanical properties of biocomposites. In this study, polypropylene random copolymer (PPRC) and biowaste rice husk (BRH) were used as the main raw materials, and glass-fiber-reinforced polypropylene and talc were also used with BRH to enhance the mechanical characterization of the biocomposites. The interfacial bonding between the polymer and the rice husk was increased by treating them with maleic anhydride and NaOH, respectively. The results obtained from the nanoindentation indicated that the plastic behavior of the biocomposites was prominent when untreated BRH was used and vice versa. The modulus and hardness of the biocomposite improved by 44.8% and 54.8% due to the neat PPRC, respectively. The tribological properties were studied based on the hardness-to-modulus ratio and it was found that BRH- and talc-based biocomposites were better than other samples in terms of low friction and wear rate. The creep measurements showed that untreated rice husk biocomposite exhibited high resistance to load deformation.

## 1. Introduction

Polypropylene random copolymer (PPRC) is a well-known recyclable thermoplastic polymer with improved mechanical properties compared to other grades of polypropylene (PP). The chain structure of PP can be controlled by the copolymerization of ethylene with other α-olefins, resulting in different types of copolymer (PP block copolymer and PPRC). The random arrangement of copolymer chains is achieved by introducing ethene (E) into PP chains. This process can result in the occurrence of different triad assemblies, i.e., EPP, PEP, EPE [1]. PP and its copolymers have applications varying from packaging (flexible and rigid), household items, parts of automobiles, industrial chemical storage tanks, piping industry, and the furniture industry. In this study, we focus on PPRC, which has low lamellar thickness and crystallinity; because of the irregular polypropylene crystallization sequence formed by the random insertion of ethylene co-units [2]. Consequently, it exhibits good transparency, moderate strength, good toughness, excellent chemical resistance, UV degradation resistance, and good weatherability. These properties mark PPRC out from other polyolefins, and it is widely used in high-performance applications where harsh environments and tribological properties are concerned.

Polymers show elastic–plastic behavior when load is applied to them as a function of time and temperature. This unwanted behavior limits their application in structural, load-bearing, and temperature-dependent applications. These properties can be improved by incorporating the polymer matrix with fibrous material. An enormous volume of research has been undertaken to discuss the advantages and disadvantages of the use of synthetic or natural fibers as reinforcing materials [3,4,5]. However, the latest environmental focus is attracting researchers to use biowaste as a source of fibrous materials as compared to synthetic materials. This will exert a twofold effect on society: (a) the damping of biowaste for useful purposes, and (b) the production of biocomposites [6,7,8,9,10,11,12,13].

Biowaste is defined as the non-edible part of crop residue. Bensten et al. studied worldwide biowaste production from six main crops (sugar cane, soyabean, rice, maize, and barley) and estimated it to be around 3.7 Pg dry matter/year, with increment of 1.3 Pg dry matter/year [14]. In general, biowastes are used for the production of electricity energy by direct or indirect pyrolysis/gasification/combustion [15], resulting in ash production, harmful gas emission, and environmental pollution [16]. This study concentrates on the use of biowaste rice husk (BRH) as a source of the fiber part of biocomposites. BRH consists of cellulose (41–28%), hemicellulose (27–22%), lignin (23–10%), and ash (24–10%) [17]. About 140 million tons/year of BRH are produced globally and the non-utilization of this huge amount leads to open burning, thus creating a negative effect on the environment [18].

The combination of wood/biowaste with polymer is generally termed wood polymer biocomposite (WPC), and it is synthesized by melt-mixing the matrix phase with the fiber phase following compression molding [19,20,21], which, upon solidification, interlocks the fiber inside the matrix phase. This interfacial bonding between matrix and fiber plays an important role in establishing the mechanical properties of WPC. Therefore, different types of compatibilizer are used to increase the interfacial activities of polymer chains [22]. In contrast, fiber is pretreated (using plasma, alkali, acid, hot-water, ionic, and biological methods) to increase its reactivity. Here, maleic anhydride (MA) was used as a compatibilizer, and the alkali treatment of BRH was used for the synthesis of WPC. Nirmal et al. and others used NaOH for the pretreatment of biowaste (kenaf fiber, rice husk, flax fiber, wheat straw) at different conditions and concluded that alkali treatment increased the composition of cellulose by removing lignin and hemicellulose [6,13,23,24,25]. Cellulosic fiber has a high elastic modulus; thus, it can enhance the elastic modulus in WPC. Ren et al. tuned the elasto-mechanical properties of PPRC by introducing different compositions of elastomer [26]. Boonsuk et al. used treated rice husk with cassava starch and reported a 220% increase in tensile strength [6]. Glass fibers are used to reinforce polymer to achieve high thermo-mechanical properties. Therefore, to further increase surface stiffness and enhance hardness, polypropylene reinforced with 30% glass fiber was used [7,27]. This multi-component wood polymer composite has particles of different sizes with different orientations. Therefore, its porosity will increase, resulting in low hardness and a high creeping effect. Furthermore, because automobile and structural applications of WPCs use talc fillers to create smooth surfaces and weathering shields and to lower the cost of production [8,19,28,29,30,31,32], talc was used as a filler.

WPCs are potentially growing synthetic materials due to their advanced mechanical properties, thermal degradation, fungal decay, mouldability, impact bearing, fire resistance, and environmental durability compared to naturally occurring wood [33,34,35,36,37,38]. Their processing is difficult and the tuning of specific properties may require extra energy and technical skills [38]. All these factors make WPC cost three-to-five times more than wood [39]. Therefore, it is challenging to replace wood in homes and the industrial sector. However, the environmental issues caused by tree cutting have increased the demand for WPC in the construction, automotive, and industrial sectors [40,41,42,43]. The surface mechanical properties of WPC are difficult to control and to study due to the dimensional instability of the ligno-cellulosic part of BRH; WPC therefore exhibits anisotropic behavior [10]. Conventional techniques measure wholesome mechanical properties; thus, they cannot identify defects in structure, elastic–plastic behavior, or the homogenous mixing of composites. Further, they exhibit limitations in test resolution, experimental conditions, and sample size [44]. Nanoindentation is a powerful, non-destructive tool to calculate mechanical properties. It is sensitive to filler dispersion, filler content, and the interfacial adhesion of matrix-fiber phases; thus, it allows the direct comparison between neat polymers and their composites [45,46,47,48]. The heterogeneities of WPC that appears due to nonhomogeneous distribution can also be detected by this technique [4]. It is extensively used to determine the variations in hardness, modulus, and creep of different systems, such as coatings, polymers, composites, metals, and alloys [7,49,50,51,52,53,54,55,56]. Technological developments, from depth-sensing indentation (DSI) to quasi-continuous stiffness measurement (QCSM), have made nanoindentation a tool to study even the bulk mechanical properties of materials [57].

To the best of our knowledge, PPRC had not been used previously to produce biocomposites. Further, the combination of treated and untreated BRH, along with glass-fiber-reinforced polymer and talc, made this research work unique. In this study, nanoindentation was used to study the surface-mechanical properties of WPC synthesized by using different compositions of raw materials. A comparative study of neat PPRC and WPC helped to understand the interfacial behavior of raw materials. The potential application of synthesized WPC is to replace tree-wood in structural applications, hence, to protect the environment and save trees.

## 2. Materials and Biocomposite Fabrication

### 2.1. Raw Materials and Reagents

Compression-grade polypropylene random copolymer (trade name R200P, melt index 0.25 g/10 min at 230 °C, with constant load 2.16 kg and density 0.90 g/cm^3^), was purchased from Topiline, Seoul, Korea and used without pretreatment. Polypropylene reinforced with 30% glass fiber (PPGF, trade name KPG1030, and density 1.14 g/cm^3^) was purchased from Kopla Co., Ltd., Hwaseong, Korea. Maleic anhydride (MA) (98%) was purchased from Unichem, India. Biowaste rice husk (BRH) was collected from local rice industries, Muridkay, Pakistan. NaOH (99%) was purchased from Sigma Aldrich, USA. Talc powder (TLC) was purchased from local supplier, Lahore, Pakistan.

### 2.2. Biomass Preparation and Pretreatment

**Figure 1:**
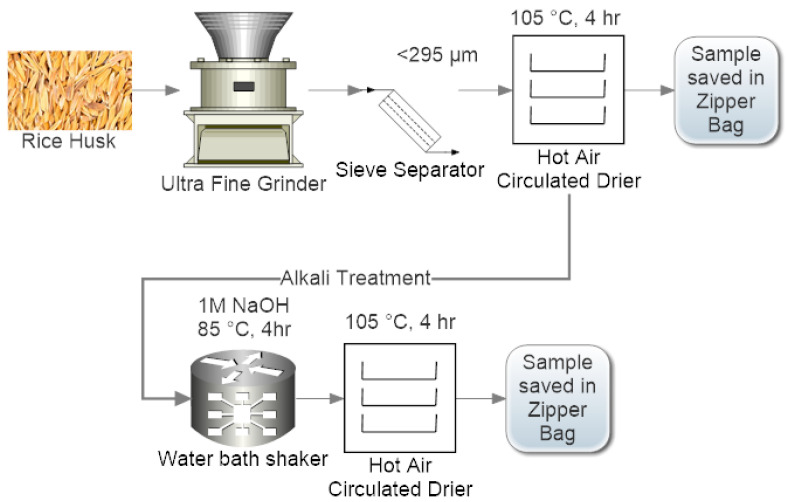
Biowaste rice husk pretreatment (untreated: top sequence, treated: bottom sequence).

BRH was cleaned from dust and stones, crushed in ultrafine grinder and sieved with a 48-mesh US-Tayler sieve; particles with diameters less than 295 um were collected (Figure 1). The sieved particles were then dried in a tray drier at 105 °C for 4 h and stored in a zipper bag for future use. Further, BRH powder was alkali-treated (BRHT) to activate surfaces of raw biomass with -OH functional group. A 10% w/v BRH was soaked in 1-molar NaOH solution and placed in a hot water bath for 4 h at 80 °C under mild oscillations. After a given time, the sample was cooled, filtered, rinsed with excess distilled water, dried for 4 h at 105 °C, and stored in zipper bags for future use.

### 2.3. Fabrication of Biocomposite

**Figure 2:**
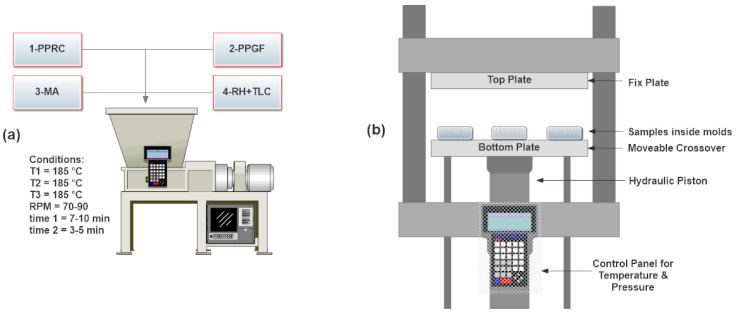
Fabrication of biocomposite (**a**) melt mixing, (**b**) thermal hydraulic compression molding.

A twin-screw batch internal mixer (Banbury internal mixer, model SBI-35L, Well Shyang Machinery Co., Ltd., Taiwan) was used to make WPC (Figure 2a). It consisted of a melt mixing chamber that had three temperature zones and two helical mixers. The temperatures of three stages of melt zone were set to 180–190 °C and axial mixer speed was fixed to 90 rpm. Initially, granules of PPRC were added into a preheated mixer until melting was observed. Next, PPGF and MA were added and mixed for 7–10 min to form a homogeneous blend. Finally, BRH (untreated or treated) and TLC were added and mixed for 3–5 min. After completion of compounding, the semi-solid biocomposite was removed, inserted into molds, and compressed in hydraulic thermal press (hydraulic platen press, Hartek Technologies Ltd., Guangzhou, China) operating at 190 °C and 2000 kPa for 10 min (Figure 2b). Next, the heaters were tuned off and samples were cooled under compression force until they reached room temperature. The prepared samples were removed from molds and stored for further testing. Table 1 indicates the composition in percentage of samples.

## 3. Nanoindentation Tests

The nanoindentation tests were performed using a noanoindenter KG-ULM from Zwick GmbH & Co. KG, Ulm, Germany. Berkovich three-sided pyramidal diamond indenter tips was used in all tests. Experiments were performed at room temperature (25 °C) under load-control mode. Quasi-continuous stiffness method (QCSM) was adopted with multi-point indentations. Distance between indents was fixed to 100 µm. This distance was selected to prevent pile-up effect during continuous indentations. An optimum retention time was selected to diminish viscous effects on the discharging portion of test specimen (without nose). Creep behavior of biocomposite was studied based on indentation holding time.

Different samples of biocomposite were cut according to sample holder dimensions and loaded for testing. The nanoindentation tests were executed at peak load of 100 mN and 20 s holding time. The standard test computing system continuously recorded testing materials’ hardness and modulus behavior.

## 4. Nanoindentation Characterization

**Figure 3:**
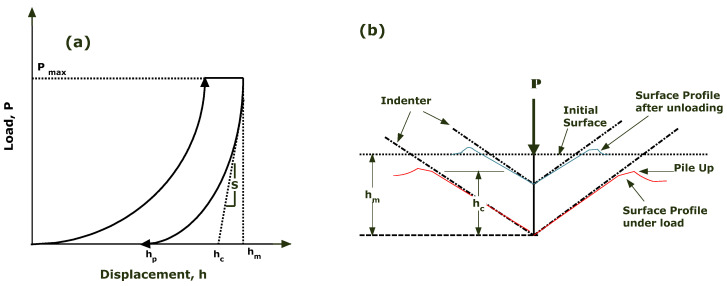
(**a**) Load–depth curve for nanoindentation of viscoelastic material. (**b**) Demonstration of the cross-section of the indenter tip during the loading–unloading portion.

The compliance method is used to analyze data produced by depth-sensing indentation, DSI, or continuous stiffness measurement, CSM, in which the preliminary section of the unloading curve is analyzed [58]. A typical load–depth curve is shown in Figure 3. The curve profile is analyzed by considering the elastic contact between the surface and the indenter. The aim of this approach is to study the hardness and elastic modulus of thermoplastic polymers when the indent stays in contact with the specimen during two-thirds of the unloading response. This condition may not be true for all types of material [55].

The maximum depth of penetration of a Berkovich indenter is $h_{m}$ when subjected to maximum load, P_max_. The contact stiffness S is calculated by taking the slope between 90% and 40% of the unloading curve dP/dh (Figure 3a), and it can be localized as pile-up around the indentation area (Figure 3b). The extrapolation of the unloading curve to zero-load is considered as plastic depth $h_{p}$. The value $h_{c}$ is the intercept of the unloading curve from P_max_ to the displacement axis, and it is also known as elastic deformation [59]. The relationship between S, the projected contact area of the Berkovich indenter A, and the reduced modules E_r_ is proposed as:

(1) $$E_{r}=\frac{\sqrt{\pi }}{2\beta }\;\frac{S}{\sqrt{A}}$$

where β = 1.034 for the Berkovich tip and *A* is the depth of the indenter tip that penetrates into material surface [60]. Doerner and Nix simplified the relationship between A and plastic depth $h_{c}$ as [58]:

(2) $$A=25.5{\;h}_{c}^{2}$$

whereas $h_{c}$ is estimated graphically from the deflection point of the P-h curve.

The estimated Poisson’s ratio is used to calculate the modulus E by incorporating $E_{r}$ and $E_{i}$ as:

(3) $$\frac{1}{E_{r}}=\frac{\left(1-v^{2}\right)}{E}+\frac{\left(1-v_{i}^{2}\right)}{E_{i}}$$

or

(4) $$E=\frac{\left(1-v^{2}\right)}{\left(\frac{1}{E_{r}}-\frac{\left(1-v_{i}^{2}\right)}{E_{i}}\;\right)}$$

where $E_{i}$ and $v_{i}$ are the indenter modulus and Poisson’s ratio, respectively, and E and v are the modulus and Poisson’s ratio of the sample, respectively. For diamond tip, $E_{i}=1141\;\mathrm{GPa}$, and $v_{i}=0.07$. For polypyrene random copolymer (PPRC), v=0.43. To calculate the biocomposite modulus E, QCSM is used to obtain a loading–unloading curve vs depth.

Oliver and Pharr’s method (OP) is widely used currently to calculate the elastic–plastic behavior of materials. In this method, the hardness H and maximum applied load $P_{\max}$ are correlated as follows:

(5) $$H=\frac{P_{\max}}{A}$$

Polymeric materials exhibit higher hardness values at the start of indentation, as the indenter tips face surface stiffness and tension [3,61,62]. Gradually, the hardness decreases until it reaches a uniform value. Many researchers call this phenomenon an error in the indentation process, and it can be minimized by proper calibration of the indenter [3,61,63,64].

## 5. Results and Discussion

### 5.1. Load Displacement Curve Study

**Figure 4:**
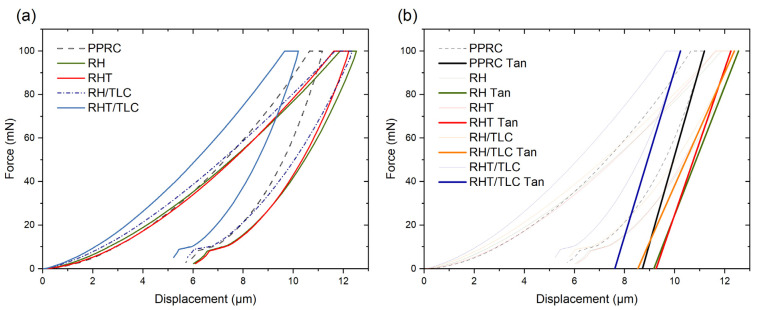
QCSM data (**a**) load–depth curve, (**b**) tangent drawn on unloading curves to calculate ${\;h}_{r}$.

Figure 4 illustrates the QCSM data obtained in the form of load–depth curves for the PPRC and different biocomposite sheets. The curves were continuous, thus indicating no discontinuity during data collection and the homogeneity of all the samples. The loading portion of all the curves began from zero until it reached $P_{\max\;}\;$= 100 mN. After unloading, the curves ended between 5 and 6 µm. This nonzero depth $h_{p}$ of the indented surface after the removal of load indicated that the composite sheets exhibited plastic behavior. From Figure 4a, it was also found that the $h_{p}$ for PPRC was 5.71 µm, whereas the biocomposite made from RHT exhibited a maximum $h_{p}$ of 6.08 µm. This increase in plastic behavior was due to the alkali treatment of RH as it increased the percentage of cellulose in the biocomposite [11,26]. On the other hand, the $h_{p}$ value was decreased to 5.22 µm by adding talc in biocomposite. This result indicated that the incorporation of filler particles in the biocomposite created good binding and dispersion, as suggested by Barun et al. [3]. To further investigate the compositional behavior of the materials, a tangent was drawn on each curve, starting from the unloading curve until it reached zero load condition. The depth found by the tangent is represented by $h_{r}$ (Figure 4b) and is known as elastic deformation [54]. This phenomenon is common in polymers. Here, higher values of elastic deformation were observed in the samples that did not have talc filler. This could have been due to interfacial adhesion, voids in the biocomposite, or the displacement of the matrix layers during indentation. The maximum depth h_m_ of the Berkovich indenter was measured as corresponding to the peak load P_max_ on the unloading curve. The PPRC demonstrated an indentation of 11.18 µm, whereas this value decreased by 1.14% for the RHT/TLC and increased by 1.40% for the RH. The contact stiffness S was calculated by dP/dh for the unloading curve and the results showed that the maximum S was found to be 40.51 mN/µm for the virgin PPRC. When pure rice husk was added to the biocomposite, the surface indentation stiffness decreased to 29.54 mN/µm and 25.9 mN/µm for the RH and RH/TLC, respectively. This reduction in S could have been due to the weak bonding between the RH and the PPRC. For the treated rice husk, the S values increased to 33.53 mN/µm and 37.8 mN/µm for the RHT and RHT/TLC, respectively, indicating strong bonding between the matrix and fiber phases [65]. The results are shown in Table 2.

### 5.2. Elastic Modulus and Hardness

**Figure 5:**
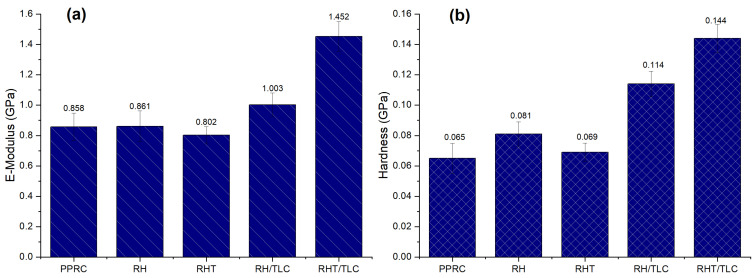
(**a**) Elastic modulus, E, and (**b**) hardness of different samples.

The elastic modulus values were obtained by using the OP method and are graphically reparented in Figure 5a. Equation (4) illustrates that E depends on Er, which is a function of surface stiffness, S, and area of indentation, A. The results indicated that the compounding of rice husk in the PPRC did not introduce a prominent effect in E when the loading of the filler was 5% (samples RH and RHT). On the other hand, the E values were enhanced by many folds by incorporating 2% talc into the biocomposites (samples RH/TLC and RHT/TLC). A close investigation of Equations (1)–(3) and Table 2 revealed that the surface stiffness S was high for the PPRC and reduced in all the biocomposite samples. The elastic deformation $h_{r}$ showed increasing behavior in samples RH and RHT and decreasing behavior in samples RH/TLC and RHT/TLC. Therefore, it can be deduced that talc elevated the resistance to deformation and, thus, E increased. The maximum E was obtained for the RHT/TLC biocomposite (1.452 GPa), which was 40.9%, 40.7%, 44.8%, and 30.9% higher than the PPRC, RH, RHT, and RH/TLC respectively.

The hardness of the synthesized samples was calculated by using Equation (5). The QCMS module directly calculated the area in which the indenter penetrated inside the material surface at the maximum applied load (Figure 5b. The results indicated that the indentation hardness reduced from 0.081 GPa to 0.069 GPa for the biocomposite when pure rice husk was replaced by alkali-treated rice husk. This decrease in value was expected, as alkali treatment reduced the lignin and hemicellulose, resulting in an increase in the concentration of cellulose, which has a low hardness value [66]. Further, the RHT/TLC biocomposite exhibited the highest hardness value, 0.144 GPa, which was 20.8%, 52.1%, 43.8%, and 54.8% higher than the RH/TLC, RHT, RH, and PPRC, respectively. The filler content in samples RH and RHT and RH/TLC and RHT/TLC was the same, but H and E varied prominently. This breakthrough value could have been the result of homogenous mixing, proper dispersion, or the crosslinked bonding between the alkali-treated rice husk, polypropylene random-copolymer, glass-fiber-reinforced propylene, and talc. Moreover, the addition of rice husk flour along with talc withstood the mobility of the polymeric chains, which resulted in high hardness. On the other hand, the pure PPRC sheet did not contain any fiber, so the polymeric chains slid over each other without any resistance, and indicating a very minimal indentation hardness.

**Figure 6:**
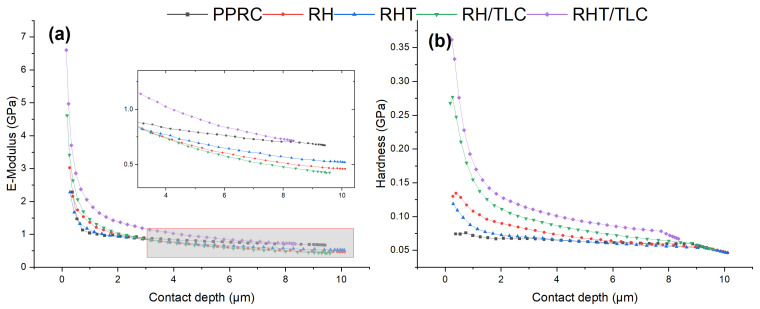
(**a**) Elastic modulus profile as a function of depth. (**b**) Hardness profile as a function of depth.

Figure 6a demonstrates the elastic modulus profiles as a function of depth. The profiles can be divided into two portions: higher values at low penetration depth (<2 µm) and constant values at high penetration depth (>2 µm). By comparing both portions, it can be concluded that at low penetration values (<2 µm), the biocomposite showed very high deviations in elastic modulus; even overlapping can be observed. Gradually, the deviations faded away and nearly constant values were obtained, which were consistent with the results presented in Figure 5a.

Figure 6b illustrates the indentation hardness profiles as a function of depth for all the sample sheets. The profiles can be divided into two portions. Firstly, higher values of hardness were obtained at low penetration and a conspicuous decrease in hardness is visible at depths ≈ 2.5 µm. This behavior indicated that the material surface hindered the indentation and, thus, exhibited high stiffness. Secondly, as the indenter travelled inside the material, its layers adjusted to or accommodated the indenter tip, which resulted in a sharp decrease in hardness. However, after 2.5 µm, the hardness variation abated due to the self-alignment of the inside layers. Sample RHT/TLC biocomposite exhibited this phenomenon visibly, but sample PPRC only demonstrated a second profile, where the hardness remained almost constant throughout the test.

### 5.3. Hardness-to-Elastic-Modulus Ratio H/E

Tribological properties play important roles in defining the performance criteria of materials. Many factors e.g., nature of raw materials, their compatibility, and addition of elastomers, control wear and tear of WPCs [67]. 

**Figure 7:**
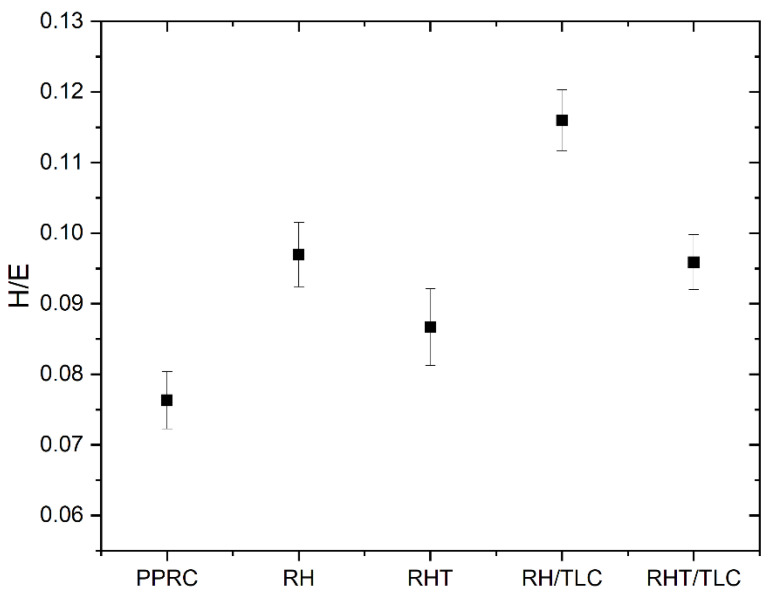
H/E ratio for different biocomposites.

The wear resistance of material, elastic strain to failure, plastic deformation under critical load, and fracture toughness can be estimated by using the H/E ratio. Leyland and Matthews predicted the wear behavior of nanocomposite coating in terms of H/E [49]. Their work provides a logical explanation of the H/E ratio, in that its higher value corresponds to low elasticity and high hardness, which makes the composite rigid and resistant to surface change, and vice versa. Later, Nikaeen et al. reported the bulk properties of low-density polyethylene (LDPE) reinforced with carbon nanofiber (CNF) and concluded, on the basis of the H/E ratio, that the wear resistance of the composite was decreased by increasing the CNF [57]. Sulaiman et al. studied LDPE biocomposites with treated rice husk and reported an increasing–decreasing trend in the H/E value corresponding to the loading of the rice husk [10]. Here, in this work, the effect of treated and untreated rice husk biocomposite was studied along with the addition of talc as a filler. Figure 7 indicates the H/E of various samples. The PPRC exhibited the lowest H/E ratio compared to the biocomposite samples. Therefore, it can be inferred that biocomposite samples offer better hardness and stiffness compared to PPRC. Further, the biocomposite samples containing untreated rice husk showed better H/E compared to those that had treated rice husk. This could have been due to the presence of high cellulose content in the treated rice husk, resulting in a high elastic modulus in the biocomposite. The maximum H/E (= 0.116) was obtained for the RH/TLC biocomposite, which was 17%, 25%, 16%, and 34% higher than that of the RHT/TLC, RHT, RH, and PPRC, respectively. This high H/E value could have been due to the presence of talc particles.

### 5.4. Creep Measurements

Metals and ceramics show deformation when load is applied at high temperatures, whereas polymer and composite deformation is also a function of time. This deformation behavior is known as creep. One of the major applications of the nanoindentation technique is in the measurement of indentation creep. In this test, a constant load is applied on the specimen and the penetration/depth of the indenter is monitored as a function of time. This method is useful compared to the conventional tensile creep test as it is sensitive to surface morphology (smooth, harsh, homogeneous, etc.) of materials [68]. Furthermore, it does not have drifting errors, resulting in the accurate observation of creep in small indents for long periods of time by measuring the mean stress and contact stiffness [69]. This technique has been used to study the creep behavior of coatings, multi-layer solids, bulk materials, polymers, and composites [59,70,71,72,73]. The depth of indentation can be measured in situ by piezoelectric depth-sensing noanoindenter. Biocomposites exhibit viscoelastic behavior due to the presence of the semi-crystalline and amorphous structures of their components.

#### 5.4.1. The Effect of Holding Time on Creep Rate

**Figure 8:**
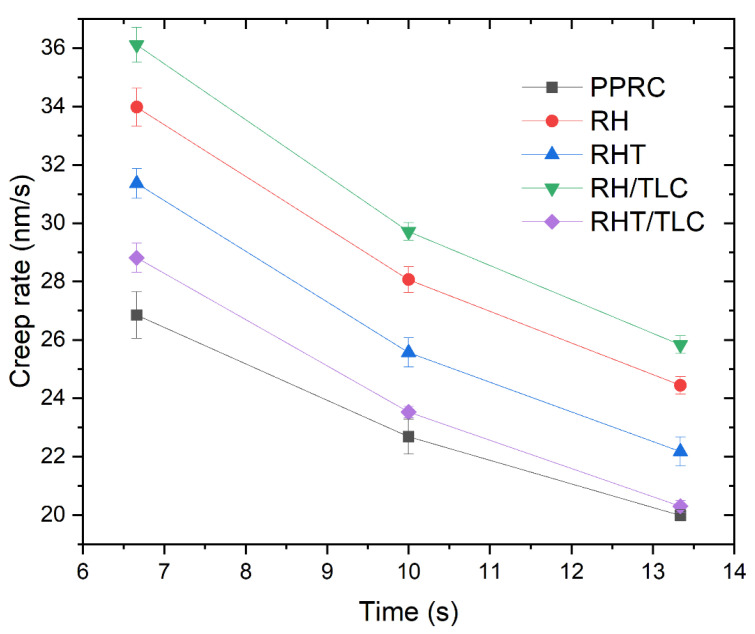
Creep rate as a function of time for different samples.

By using the QCSM method, viscoelastic behavior can be directly studied. Figure 8 represents the creep rate as a function of time. A constant peak load of 100 mN was applied on all the samples for a period of 20 s. To avoid a pileup effect during indenter holdup, the results were recorded at three different time intervals: 6.7 s, 10 s, and 13.3 s. The PPRC exhibited the lowest creep rate among all the samples, indicating its highly crystalline structure. By contrast, the biocomposite demonstrated an increase in creep rate, resulting in amorphization. The shift away from a crystalline structure was greater for the samples that contained untreated rice husk. Moreover, the gap narrowed for the samples containing treated rice husk. This could have been due to the effect of the compatibilizer, which created proper bonding between the polymer chain and the treated rice husk. Sample RHT/TLC showed the lowest creep rate among the wood polymer biocomposite samples and, at the holding time of 13.3 s, its creep rate reached a value of 20.3 nm/s (1.48% higher from PPRC). Therefore, the addition of talc particles filled the voids due to the rice husk–PPRC composite.

#### 5.4.2. The Effect of Holding Time on Depth

**Figure 9:**
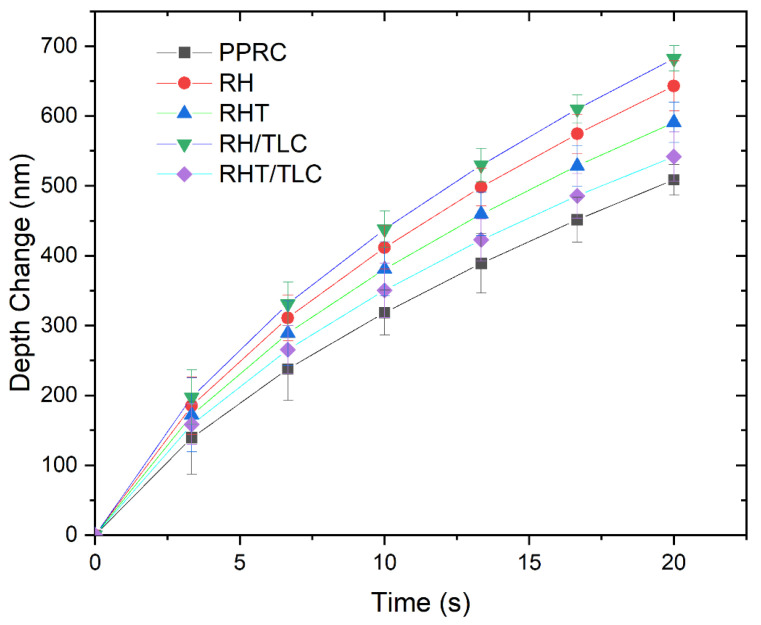
Depth change as a function of time for different samples.

The QCSM module of nanoindentation can directly record the effect of depth change as a function of time by using the OP method and keeping loads at constant values. The results are represented in Figure 9, at a peak load of 100 mN and 20 s. At the start of this test, all the samples showed a narrow range of depth change 18 nm (140–158 nm) and, as the time increased, this gap widened and ended at 174 nm (508–682 nm). The PPRC showed the lowest depth change, of 508 nm, whereas the RH/TLC demonstrated the highest depth change, of 682 nm. These results might be an indication of the compact PPRC’s crystalline structure, which was not available in the RH/TLC composite. Therefore, the further addition of talc particles filled the gaps in the fiber–matrix composite (RHT/TLC) and resulted in a rigid material, which showed a depth change of 542 nm (20% lower than the RH/TLC).

## 6. Conclusions

The mechanical properties of the PPRC were successfully enhanced by incorporating rice husk, glass fiber, and talc. Their combined effect (pre-treatment and incorporation of talc) was synergetic. The modulus of the WPC showed a minor increment of 0.858 to 0.861 GPa from the PPRC to the RH, respectively. A major increase in modulus, 1.452 GPa, was observed for the RHT/TLC, which was 40.9% more than that of the PPRC. Similarly, the highest hardness, 0.144 GPa, was obtained for the RHT/TLC biocomposite; this was 54.8% higher than that of the PPRC. On the other hand, the RHT biocomposite showed a hardness of 0.069 GPa, which was only 0.94% higher than that of the PPRC. These results indicate that although the alkali-treated rice husk increased the interfacial adhesion of the polymer and fiber phases, talc played a synergetic role as a filler. It might have filled the voids present in the WPC and created stronger interlocking in the rice husk. The behavior of the talc became more prominent when the H/E ratios of all the samples were compared. A higher H/E was obtained for the RH/TLC and RH biocomposites, making them good for rigid and resistant-to-surface-change applications. Here, the role of untreated rice husk was also important as it created stiffness on the surfaces of the composites, making them hard and less elastic. It can be concluded from this research that the adjustment of various concentrations of raw materials may result in wood polymer composites with enhanced mechanical properties. However, the effect of higher concentrations of rice husk needs to be verified through further research work. Furthermore, tribological behavior studied with the help of nanoindentation needs to be checked by a tribometer to study the wear and friction coefficient of WPC.

## Figures and Tables

**Table 1:** Composition of raw materials for wood polymer biocomposite, wt/wt%.

Sample Name	BRH	BRHT	PP	PPGF	MA	TLC
PPRC	-	-	100	-	-	-
RH	5	-	88	5	2	-
RHT	-	5	88	5	2	-
RH/TLC	5	-	86	5	2	2
RHT/TLC	-	5	86	5	2	2

**Table 2:** Values of plastic depth $h_{p},$ elastic deformation $h_{r},$ maximum penetration depth $h_{m}$, and contact stiffness S calculated by nanoindetation at P_max_ = 100 mN.

Sample	$h_{p}\;(µm)$	$h_{r}\;(µm)$	$h_{m}\;(µm)$	S (mN/µm)
PPRC	5.71	8.71	11.18	40.51
RH	6.02	9.16	12.55	29.54
RHT	6.08	9.25	12.24	33.53
RH/TLC	5.78	8.52	12.38	25.9
RHT/TLC	5.22	7.60	10.22	37.8

## Data Availability

All the supporting and actual data has already been presented in the manuscript.

## References

[1] Galli P., Haylock J.C., Simonazzi T. (1995). Manufacturing and properties of polypropylene copolymers. Polypropylene Structure, Blends and Composites.

[2] Harding G.W., Van Reenen A.J. (2006). Fractionation and Characterisation of Propylene-Ethylene Random Copolymers: Effect of the Comonomer on Crystallisation of Poly(propylene) in the γ-Phase. Macromol. Chem. Phys..

[3] Das B., Eswar Prasad K., Ramamurty U., Rao C.N.R. (2009). Nano-indentation studies on polymer matrix composites reinforced by few-layer graphene. Nanotechnology.

[4] Liu T., Phang I.Y., Shen L., Chow S.Y., Zhang W. (2004). De Morphology and Mechanical Properties of Multiwalled Carbon Nanotubes Reinforced Nylon-6 Composites. Macromolecules.

[5] Detrez F., Cantournet S., Seguela R. (2011). Plasticity/damage coupling in semi-crystalline polymers prior to yielding: Micromechanisms and damage law identification. Polymer.

[6] Boonsuk P., Sukolrat A., Bourkaew S., Kaewtatip K., Chantarak S., Kelarakis A., Chaibundit C. (2021). Structure-properties relationships in alkaline treated rice husk reinforced thermoplastic cassava starch biocomposites. Int. J. Biol. Macromol..

[7] Perna A.S., Astarita A., Carlone P., Guthmann X., Viscusi A. (2021). Characterization of cold-spray coatings on fiber-reinforced polymers through nanoindentation tests. Metals.

[8] Guna V., Ilangovan M., Rather M.H., Giridharan B.V., Prajwal B., Vamshi Krishna K., Venkatesh K., Reddy N. (2020). Groundnut shell/rice husk agro-waste reinforced polypropylene hybrid biocomposites. J. Build. Eng..

[9] Konstantopoulos G., Semitekolos D., Koumoulos E.P., Charitidis C. (2021). Carbon fiber reinforced composites: Study of modification effect on weathering-induced ageing via nanoindentation and deep learning. Nanomaterials.

[10] Sulaiman M., Iqbal T., Yasin S., Mahmood H., Shakeel A. (2020). Study of nano-mechanical performance of pretreated natural fiber in ldpe composite for packaging applications. Materials.

[11] Soleimani M., Tabil L., Panigrahi S., Opoku A. (2008). The effect of fiber pretreatment and compatibilizer on mechanical and physical properties of flax fiber-polypropylene composites. J. Polym. Environ..

[12] Abu Bakar M.B., Mohd Ishak Z.A., Mat Taib R., Rozman H.D., Mohamad Jan S. (2010). Flammability and mechanical properties of wood flour-filled polypropylene composites. J. Appl. Polym. Sci..

[13] Bisht N., Gope P.C. (2019). Wear Characteristics of Untreated and Alkali-Treated Rice Husk–Epoxy Bio-composite. Trends in Materials Engineering.

[14] Bentsen N.S., Felby C., Thorsen B.J. (2014). Agricultural residue production and potentials for energy and materials services. Prog. Energy Combust. Sci..

[15] Mohiuddin O., Mohiuddin A., Obaidullah M., Ahmed H., Asumadu-Sarkodie S. (2016). Electricity production potential and social benefits from rice husk, a case study in Pakistan. Cogent Eng..

[16] Suhot M.A., Hassan M.Z., Aziz S.A., Md Daud M.Y. (2021). Recent progress of rice husk reinforced polymer composites: A review. Polymers.

[17] Korotkova T.G., Ksandopulo S.J., Donenko A.P., Bushumov S.A., Danilchenko A.S. (2016). Physical Properties and Chemical Composition of the Rice Husk and Dust. Orient. J. Chem..

[18] Bakker R., Elbersen W., Lesschen P. (2013). Rice Straw and Wheat Straw. Potential Feedstocks for the Biobased Economy.

[19] Hidalgo-Salazar M.A., Salinas E. (2019). Mechanical, thermal, viscoelastic performance and product application of PP- rice husk Colombian biocomposites. Compos. Part B Eng..

[20] Bisht N., Gope P.C., Rani N. (2020). Rice husk as a fibre in composites: A review. J. Mech. Behav. Mater..

[21] Nizamuddin S., Jadhav A., Qureshi S.S., Baloch H.A., Siddiqui M.T.H., Mubarak N.M., Griffin G., Madapusi S., Tanksale A., Ahamed M.I. (2019). Synthesis and characterization of polylactide/rice husk hydrochar composite. Sci. Rep..

[22] Ambrogi V., Carfagna C., Cerruti P., Marturano V. (2017). Additives in Polymers. Modification of Polymer Properties.

[23] Li X., Panigrahi S., Tabil L.G. (2009). A study on flax fiber-reinforced polyethylene biocomposites. Appl. Eng. Agric..

[24] Nirmal U., Lau S.T.W., Hashim J. (2014). Interfacial Adhesion Characteristics of Kenaf Fibres Subjected to Different Polymer Matrices and Fibre Treatments. J. Compos..

[25] Karthigairajan M., Nagarajan P.K., Raviraja Malarvannan R., Ramesh Bapu B.R., Jayabalakrishnan D., Maridurai T., Shanmuganathan V.K. (2021). Effect of Silane-Treated Rice Husk Derived Biosilica on Visco-Elastic, Thermal Conductivity and Hydrophobicity Behavior of Epoxy Biocomposite Coating for Air-Duct Application. Silicon.

[26] Ren Q., Fan J., Zhang Q., Yi J., Feng J. (2016). Toughened polypropylene random copolymer with olefin block copolymer. Mater. Des..

[27] Sathishkumar T.P., Satheeshkumar S., Naveen J. (2014). Glass fiber-reinforced polymer composites—A review. J. Reinf. Plast. Compos..

[28] Monti M., Scrivani M.T., Gianotti V. (2020). Effect of SEBS and OBC on the impact strength of recycled polypropylene/talc composites. Recycling.

[29] Singh T., Gangil B., Patnaik A., Biswas D., Fekete G. (2019). Agriculture waste reinforced corn starch-based biocomposites: Effect of rice husk/walnut shell on physicomechanical, biodegradable and thermal properties. Mater. Res. Express.

[30] Jiang H., Kamdem D.P. (2004). Development of poly(vinyl chloride)/wood composites. A literature review. J. Vinyl Addit. Technol..

[31] Satov D. (2008). Additives for wood polymer composites. Wood-Polymers Composites.

[32] Tolinski M. (2015). Coupling and Compatibilizing. Additives for Polyolefins.

[33] Wood Plastic Composite Market by Type & Application—Global Forecast 2021|MarketsandMarkets|Last Updated on July-2020. Marketsandmarkets (2016). https://www.marketsandmarkets.com/Market-Reports/wood-plastic-composite-market-170450806.html.

[34] Moy S. (2013). Advanced fiber-reinforced polymer (FRP) composites for civil engineering applications. Developments in Fiber-Reinforced Polymer (FRP) Composites for Civil Engineering.

[35] Ning C., Zhou L., Tan G. (2016). Fourth-generation biomedical materials. Mater. Today.

[36] Vadivelu M.A., Kumar C.R., Joshi G.M. (2016). Polymer composites for thermal management: A review. Compos. Interfaces.

[37] Tripathi G., Choudhury P., Basu B. (2010). Development of polymer based biocomposites: A review. Mater. Technol..

[38] Gardner D.J., Han Y., Wang L. (2015). Wood–Plastic composite technology. Curr. For. Rep..

[39] KLYOSOV A.A. (2007). Wood-Plastic Composites.

[40] Wang J. (2013). Assessing the durability of the interface between fiber-reinforced polymer (FRP) composites and concrete in the rehabilitation of reinforced concrete structures. Developments in Fiber-Reinforced Polymer (FRP) Composites for Civil Engineering.

[41] Ashori A. (2008). Wood-plastic composites as promising green-composites for automotive industries!. Bioresour. Technol..

[42] Smith P.M., Wolcott M.P. (2006). Opportunities for wood/natural fiber-plastic composites in residential and industrial applications. For. Prod. J..

[43] Uddin N., Vaidya A., Vaidya U., Pillay S. (2013). Thermoplastic composite structural insulated panels (CSIPs) for modular panelized construction. Developments in Fiber-Reinforced Polymer (FRP) Composites for Civil Engineering.

[44] Phani P.S., Oliver W.C., Pharr G.M. (2021). Measurement of hardness and elastic modulus by load and depth sensing indentation: Improvements to the technique based on continuous stiffness measurement. J. Mater. Res..

[45] Kim J.K., Sham M.L., Wu J. (2001). Nanoscale characterisation of interphase in silane treated glass fibre composites. Compos. Part A Appl. Sci. Manuf..

[46] Yedla S.B., Kalukanimuttam M., Winter R.M., Khanna S.K. (2008). Effect of shape of the tip in determining interphase properties in fiber reinforced plastic composites using nanoindentation. J. Eng. Mater. Technol. Trans. ASME.

[47] Hardiman M., Vaughan T.J., McCarthy C.T. (2017). A review of key developments and pertinent issues in nanoindentation testing of fibre reinforced plastic microstructures. Compos. Struct..

[48] Kumaravel D., Mohanraj B., Sivaraj M. (2018). Enhancing the property of SS316 steel using polyether ether ketone. Mater. Sci. Eng..

[49] Leyland A., Matthews A. (2000). On the significance of the H/E ratio in wear control: A nanocomposite coating approach to optimised tribological behaviour. Wear.

[50] Sun H., Yang X., Wei K. (2019). Non-isothermal crystallization kinetics of continuous glass fiber- reinforced poly (ether ether ketone) composites. J. Therm. Anal. Calorim..

[51] Mclauchlin A.R., Ghita O.R., Savage L. (2014). Studies on the reprocessability of poly (ether ether ketone) (PEEK). J. Mater. Process. Technol..

[52] Prasad K.E., Das B., Maitra U., Ramamurty U., Rao C.N.R. (2009). Extraordinary synergy in the mechanical properties of polymer matrix composites reinforced with 2 nanocarbons. Proc. Natl. Acad. Sci. USA.

[53] Samad M.A., Sinha S.K. (2011). Mechanical, thermal and tribological characterization of a UHMWPE film reinforced with carbon nanotubes coated on steel. Tribol. Int..

[54] Arora G., Pathak H. (2021). Nanoindentation characterization of polymer nanocomposites for elastic and viscoelastic properties: Experimental and mathematical approach. Compos. Part C Open Access.

[55] Mokhtari A., Tala-Ighil N., Masmoudi Y.A. (2021). Nanoindentation to Determine Young’s Modulus for Thermoplastic Polymers. J. Mater. Eng. Perform..

[56] Kiang C.-H., Goddard W.A., Beyers R., Bethune D.S., Ajayan P.M. (1995). Nanotubes from Carbon. Chem. Rev..

[57] Nikaeen P., Depan D., Khattab A. (2019). Surface mechanical characterization of carbon nanofiber reinforced low-density polyethylene by nanoindentation and comparison with bulk properties. Nanomaterials.

[58] Doerner M.F., Nix W.D. (1986). A method for interpreting the data from depth-sensing indentation instruments. J. Mater. Res..

[59] Fischer-Cripps C. (2004). Anthony, Nanoindentation.

[60] Tranchida D., Piccarolo S., Loos J., Alexeev A. (2007). Mechanical Characterization of Polymers on a Nanometer Scale through Nanoindentation. A Study on Pile-up and Viscoelasticity. Macromolecules.

[61] VanLandingham M.R., Villarrubia J.S., Guthrie W.F., Meyers G.F. (2001). Nanoindentation of polymers: An overview. Macromol. Symp..

[62] Díez-Pascual A.M., Gómez-Fatou M.A., Ania F., Flores A. (2015). Nanoindentation in polymer nanocomposites. Prog. Mater. Sci..

[63] Zou H., Wu S., Shen J. (2008). Polymer/Silica Nanocomposites: Preparation, Characterization, Properties, and Applications. Chem. Rev..

[64] Sanchez C., Julián B., Belleville P., Popall M. (2005). Applications of hybrid organic–inorganic nanocomposites. J. Mater. Chem..

[65] Mahmood H., Moniruzzaman M., Yusup S., Muhammad N., Iqbal T., Akil H.M. (2017). Ionic liquids pretreatment for fabrication of agro-residue/thermoplastic starch based composites: A comparative study with other pretreatment technologies. J. Clean. Prod..

[66] Cruz N., Cecilia B., María G.A., Alain C., Rosario C. (2018). Impact of the Chemical Composition of Pinus radiata Wood on its Physical and Mechanical Properties Following Thermo-Hygromechanical Densification. BioResources.

[67] Mazzanti V., Fortini A., Malagutti L., Ronconi G., Mollica F. (2021). Tribological Behavior of a Rubber-Toughened Wood. Polymers.

[68] Syed Asif S.A., Pethica J.B. (2011). Nano Scale Creep and the Role of Defects. MRS Online Proc. Libr..

[69] Li X., Bhushan B. (2002). A review of nanoindentation continuous stiffness measurement technique and its applications. Mater. Charact..

[70] Ahmad A., Mansor N., Mahmood H., Iqbal T., Moniruzzaman M. (2021). Effect of ionic liquids on thermomechanical properties of polyetheretherketone-multiwalled carbon nanotubes nanocomposites. J. Appl. Polym. Sci..

[71] Sneddon I.N. (1965). The relation between load and penetration in the axisymmetric boussinesq problem for a punch of arbitrary profile. Int. J. Eng. Sci..

[72] Pethica J.B., Oliver W.C. (1988). Mechanical Properties of Nanometre Volumes of Material: Use of the Elastic Response of Small Area Indentations. MRS Online Proc. Libr..

[73] Li X., Bhushan B. (2000). Development of continuous stiffness measurement technique for composite magnetic tapes. Scr. Mater..

